# Influences of socioeconomic factors on childhood and adolescent overweight by gender in Korea: cross-sectional analysis of nationally representative sample

**DOI:** 10.1186/1471-2458-14-324

**Published:** 2014-04-07

**Authors:** Jin-Won Noh, Young-eun Kim, In-Hwan Oh, Young Dae Kwon

**Affiliations:** 1 Department of Healthcare Management, Eulji University, 212 Yangji-dong, Sujeong-gu, Seongnam-si, Gyeonggi-do 461-713, Korea; 2 Department of Biostatistics, Korea University College of Medicine, Anam-dong, Seongbuk-gu, Seoul 136-705, Korea; 3 Department of Preventive Medicine, School of Medicine, Kyung Hee University, 26 Kyungheedae-ro, Dongdaemun-gu, Seoul 130-701, Korea; 4 Department of Humanities and Social Medicine, College of Medicine and Catholic Institute for Healthcare Management, the Catholic University of Korea, 222 Banpo-daero, Seocho-gu, Seoul 137-701, Korea

**Keywords:** Childhood and adolescent overweight, Korea, Socioeconomic factors

## Abstract

**Background:**

Childhood and adolescent overweight is a recognized public health concern as the prevalence is already high and continues to increase. The purpose of this study was to examine the relationship between socioeconomic status (SES) and overweight status by gender among Korean children and adolescents.

**Methods:**

The data used in this study were taken from the 2009 Korean Survey on the Obesity of Youth and Children. Underweight individuals (*n* = 1,010) and children and adolescents whose age, height, or weight information was missing (*n* = 591) were excluded from the data set, resulting in a total of 8,555 subjects who were included in this analysis. Subjective SES, parental education level, parental occupational status, and family structure were used to measure parental SES. Chi-squared tests were used for univariable analysis and multiple logistic regression analysis was conducted for multivariable analysis.

**Results:**

After adjusting for subject’s characteristics including gender, age, parental interest in weight management of children, parental body shape, economic status variables that significantly influenced childhood overweight were identified. Low economic status increased the probability of childhood overweight (odds ratio, 1.3; 95% confidence interval, 1.1-1.5).

**Conclusions:**

There is an inverse association between parental SES variables and the overweight status of children and adolescents. Additionally, parental body shape is an important factor that influences childhood and adolescent overweight.

## Background

Overweight in children and adolescents is considered to be a significant global public health concern as the prevalence is already high and continues to increase. As shown in recent studies, during the past 30 years, overweight had more than doubled in children and tripled in adolescents [1,2]. In 2010, over one third of children and adolescents were obese [2]. Similarly, from 1997 to 2005, the prevalence of overweight among all children and adolescents increased by 70% in South Korea [3]. These figures are alarming, since overweight heightens the risk of adverse physical health outcomes, causes negative psychosocial consequences, and increases the likelihood of excessive weight in adulthood [4]. In this regard, the prevention of overweight in children and adolescents is essential and requires an understanding of possible causative factors.

Over the years, the influencing factors in the development of overweight, genetic, environmental, lifestyle, and socioeconomic variables have been discussed [5]. Recently, important parental characteristics that might impact childhood and adolescent overweight have been recognized as influential risk factors; namely, a combination of genetic, epigenetic, social, and environmental factors has been identified. For instance, having parents, especially mothers, who were overweight, increased the risk of children being overweight [6,7]. Children with two obese parents also had a higher risk of being obese than those with one or no obese parents [8]. In reality, with regards to the association between parental overweight and childhood overweight by gender, findings are conflicting. Some studies found different association in parental overweight between boys and girls, whereas others did not [9].

Parental socioeconomic variables are also known to increase the risk of childhood and adolescent overweight. An inverse correlation has been found between childhood overweight and parental education, occupation, and income levels in most studies since 1989 [10]. Adolescents with parents from a lower socioeconomic status (SES) have poorer diets and engage in less physical activity than adolescents with parents from a higher SES [11], which may partially explain this association. In addition, examination of the association between childhood overweight and SES has revealed that the inverse association between parental education and overweight is more consistent than parental occupation or income [10]. Furthermore, the SES-childhood overweight association was consistent between genders: an inverse relationship was identified [10].

Although the importance of childhood and adolescent overweight and its association with parental SES has been recognized, little research has been conducted, and most results have been inconsistent. Previous studies regarding adolescent overweight have utilized unrepresentative samples and have merely analyzed traditional socioeconomic variables such as income and education level of parents, leading to inconsistent results [12,13]. In order to develop effective prevention and treatment programs for adolescent overweight, identifying the modifiable determinants of socioeconomic variables is necessary.

The purpose of the present study is to examine the relationship between parental SES and overweight in Korean children and adolescents through analysis of nationally representative data.

## Methods

### Data source and study samples

The data utilized in this study were taken from the 2009 Korean Survey on the Obesity of Youth and Children conducted by the National Youth Policy Institute (NYPI). The entire population for this survey was nationwide students from the fourth grades in elementary school to high school. The study sample was chosen by stratified cluster sampling. The entire population was classified by the school level (elementary, middle, high school) according to the “2008 Statistical Yearbook of Education,” which was the sampling frame, and then the population was stratified by 12 administrative districts. At the first stage, schools were chosen with a selection probability proportional to the number of students in each district. At the second stage, one classroom within each of the selected schools was randomly chosen. All students within the selected classroom were eligible to participate. After obtaining a written consent from the principal of one’s school and legal guardian, face to face interviews were carried out by educated interviewers who used structured questionnaires. These interviews were conducted between June and September of 2009. The samples’ response rate was 93.9%. A total of 10,156 students participated in the survey.

We analyzed 8,555 subjects, excluding 591 children and adolescents with missing data on age, height, or weight and 1,010 individuals with low body weight since this study focused on overweight. Because this study was based on information from the 2009 Korean Survey on the Obesity of Youth and Children by the NYPI that was comprised of thoroughly deidentified secondary data released to the public for research purposes [14], this study was exempt from full review by an ethical committee at the Eulji University (EU12-18).

### Variables

Body mass index (BMI) is a widely used measure of adiposity that is calculated as weight in kilograms divided by height in meters, squared (kg/m^2^). The height and weight variables were self-reported. To define childhood overweight by BMI in this study, participants were classified using the extended International Obesity Task Force (IOTF) BMI cut-offs, which were age and gender specific cut‒off points that were extrapolated from the adult BMI cut‒offs of 25 kg/m^2^ for overweight [15]. For statistical analysis, weight status served as the dependent variable and was classified into normal weight and overweight. The underweight group was excluded in this study [6,16,17].

In this study, parental SES served as an explanatory variable that could be measured by various factors. We selected four categories to measure parental SES based on the NYPI questionnaire: subjective economic status, parental education level, parental occupational status, and family structure. To measure subjective household economic status, participants were asked, “How would you rate your household economic status on the following scale?” and responded based on a 7-point Likert scale with 1 corresponding to “very poor” and 7 corresponding to “very wealthy”. Parental education level was divided into three categories (middle school graduated or lower, high school graduated, university graduated or higher) and both the paternal and maternal education levels were included in the questionnaire. Parental occupational status was determined by whether he or she was employed, but the type of occupation was not specified. Data on family structure were obtained from demographic information in the questionnaires. Family structure was divided into four types: parents living with children, single-parent family (father), single-parent family (mother), and without parents.

We considered gender, education (age), region, parents interest in weight control and parent’s body shape as potential confounders. To measure subjective interest of parents in their children’s weight management, participants were asked “How would you rate the interest of your parents in your weight management on the following scale?” and responded based on a 5-point Likert scale with 1 corresponding to “very interested” and 5 corresponding to “no interest”. To measure subjective parental body shape, participants were asked “How would you rate your parental body shape on the following scale?” and responded along a 5-point Likert scale with 1 corresponding to “very slim” and 5 corresponding to “very heavy”. All variables were self-reported and measured by a structured questionnaire.

### Statistical analysis

For univariable analysis, the chi-squared test was used. For multivariable analysis, multiple logistic regression was applied using variables with a P value of less than 0.05 from the result of univariable analysis. Besides significant variables, father’s employment was considered to be an independent variable as well, because it is usually used as a proxy measure for economic levels. Logistic regression was used to calculate the odds ratio (OR) and 95% confidence interval (CI), while adjusting for possible confounders. And we analyzed the stratification logistic regression by gender. In order to examine the presence of multicollinearity, we confirmed the variance inflation factor and the logistic regression model didn’t show multicollinearity. All of the statistical analyses were two sided, and *P* < 0.05 was considered to be significant. For statistical analyses, SAS version 9.2 (SAS Institute, Cary, NC, USA) was used.

## Results

### General characteristics of subjects

All 8,555 subjects were composed of 4,706 males (55.0%) and 3,849 females (45.0%). Regarding education, 29.5% were elementary school students, 70.5% were middle and high school students. For economic status, 45.9% answered ‘average’, 34.6% answered ‘high’, and 19.5% answered ‘low’. 79.5% of subjects were categorized into the below normal weight zone, while 20.6% were classified as being overweight or in the obese zone. The average BMI was 20.3 with a range of 14.1 to 40.6. Among the total sample of 8,555 subjects, there were 7,362 adolescents (86.1%) living with parents. Of the adolescents residing with one parent (8.8%), 308 lived in single-father households (3.6%) and 443 in single-mother households (5.2%). The remainder of the 442 adolescents (5.2%) in the sample represented individuals who did not live with their biological parents, but resided with grandparents, relatives, and/or siblings (Table 1).

**Table 1:** Demographic characteristics of children and adolescents

**Variable**	**Category**	***n *** **(%)**
Gender	Male	4706 (55.0)
	Female	3849 (45.0)
Education	Elementary school 4th grade – 6th grade	2524 (29.5)
	Senior secondary school	6031 (70.5)
Region	Capital city (Seoul)	1417 (16.7)
	Metropolitan city	2838 (33.5)
	Cities and country	4226 (49.8)
Economic status	Low	1613 (19.5)
	Average	3790 (45.9)
	High	2863 (34.6)
Father’s education	Middle school graduated or less	425 (5.2)
	High school graduated	3401 (41.6)
	University graduated or more	4345 (53.2)
Mother’s education	Middle school graduated or less	443 (5.4)
	High school graduated	4269 (52.3)
	University graduated or more	3449 (42.3)
Father’s job	No	323 (3.9)
	Yes	7998 (96.1)
Mother’s job	No	2797 (33.6)
	Yes	5538 (66.4)
BMI	Normal weight	6797 (79.4)
	Overweight	1758 (20.6)
	Mean (±SD)	20.3 (±3.2)
	(min, max)	(14.1, 40.6)
Parental interest in weight control	Very high	3514 (41.2)
	Average	3150 (37.0)
	Little	1856 (21.8)
Father’s body shape	Slim	1900 (22.4)
	Average	3724 (43.8)
	Obese	2874 (33.8)
Mother’s body shape	Slim	1834 (21.6)
	Average	3776 (44.4)
	Obese	2897 (34.0)
Family structure	Parents living with children	7362 (86.0)
	Single-parent family (father)	308 (3.6)
	Single-parent family (mother)	443 (5.2)
	Non-parent	442 (5.2)

*BMI* body mass index; *SD* standard deviation.

### Comparison of the general characteristics of the normal weight and overweight groups

To identify differences in the characteristics of subjects that influence the level of overweight, univariable analysis was performed. This analysis found that males were more likely to be obese than females, as 26.0% of males belonged to the overweight or obese group, while females made up only 13.9% of the group (*P* < 0.0001). Students from 4th to 6th grade were more likely to be overweight than middle and high school (*P* < 0.0001). It was found that a higher proportion of students from low or high economic backgrounds belonged to the overweight group compared to students from the average economic background (*P* = 0.04). The proportion of overweight was slightly higher among the students with an unemployed father (*P* = 0.07). When parents were overweight, the children were more likely to be overweight (father’s body shape *P* < 0.0001, mother’s body shape *P* = 0.01), and as parental interest in weight management increased, the overweight status of children increased as well (*P* < 0.0001) (Table 2).

**Table 2:** Univariable analysis for childhood and adolescent overweight

**Variable**	**Category**	**Normal weight**	**Overweight**	**Total**	*P*-value^*^
Gender	Male	3482 (74.0)	1224 (26.0)	4706	< 0.0001
	Female	3315 (86.1)	534 (13.9)	3849	
Education	Elementary school 4th grade – 6th grade	1847 (73.2)	677 (26.8)	2524	< 0.0001
	Senior secondary school	4950 (82.1)	1081 (17.9)	6031	
Region	Capital city (Seoul)	1125 (79.4)	292 (20.6)	1417	0.95
	Metropolitan city	2249 (79.3)	589 (20.8)	2838	
	Cities and county	3362 (79.6)	864 (20.4)	4226	
Economic status	Low	1267 (78.6)	346 (21.5)	1613	0.04
	Average	3062 (80.8)	728 (19.2)	3790	
	High	2248 (78.5)	615 (21.5)	2863	
Father’s education	Middle school graduated or less	330 (77.7)	95 (22.4)	425	0.54
	High school graduated	2708 (79.6)	693 (20.4)	3401	
	University graduated or more	3472 (79.9)	873 (20.1)	4345	
Mother’s education	Middle school graduated or less	349 (78.8)	94 (21.2)	443	0.62
	High school graduated	3410 (79.9)	859 (20.1)	4269	
	University graduated or more	2726 (79.0)	723 (21.0)	3449	
Father employed	No	244 (75.5)	79 (24.5)	323	0.07
	Yes	6378 (79.7)	1620 (20.3)	7998	
Mother employed	No	2236 (79.9)	561 (20.1)	2797	0.50
	Yes	4392 (79.3)	1146 (20.7)	5538	
Parental interest in weight control	Very much	2449 (69.7)	1065 (30.3)	3514	< 0.0001
	Average	2607 (82.8)	543 (17.2)	3150	
	Little	1712 (92.2)	144 (7.8)	1856	
Father’s body shape	Slim	1551 (81.6)	349 (18.4)	1900	< 0.0001
	Average	3008 (80.8)	716 (19.2)	3724	
	Obese	2201 (76.6)	673 (23.4)	2874	
Mother’s body shape	Slim	1450 (79.1)	384 (20.9)	1834	0.01
	Average	3057 (81.0)	719 (19.0)	3776	
	Obese	2253 (77.8)	644 (22.2)	2897	
Family structure	Parents living with children	5868 (79.7)	1494 (20.3)	7362	0.11
	Single-parent family (father)	251 (81.5)	57 (18.5)	308	
	Single-parent family (mother)	335 (75.6)	108 (24.4)	443	
	Non-parent	343 (77.6)	99 (22.4)	442	

^*^Chi-squared test.

### Effects of SES of parents on the overweight status of children

The result of multivariable logistic regression showed that gender, education level, parental economic level, interest of parents in weight management, and body shape of father and mother were significant variables. From the analysis, it was found that the probability of students being overweight increased significantly with lower levels of parental economic level [*P* = 0.03; OR, 1.1; 95% CI, 1.0-1.3 (middle level vs. upper level) and OR, 1.3; 95% CI, 1.1-1.5 (lower level vs. upper level)] (Table 3). Education level, interest of parents in weight management, and body shape of parents were significant variables in the multivariable logistic regression to analyze the male subjects. For females, education level, parental economic level, interest of parents in weight management, and the body shape of parents were significant variables (Table 4).

**Table 3:** Adjusted odds ratio from multivariable logistic regression for childhood and adolescent overweight

**Variable**	**Category**	**OR**	**95% CI**	***P*-value**
Gender	Female	1.0		< .0001
	Male	2.9	(2.5, 3.2)	
Education	Senior secondary school	1.0		< .0001
	Elementary	1.8	(1.6, 2.0)	
Economic status	Upper	1.0		0.03
	Middle	1.1	(1.0, 1.3)	
	Lower	1.3	(1.1, 1.5)	
Father employed	Yes	1.0		0.08
	No	1.3	(1.0, 1.7)	
Parental interest in weight	Very much	1.0		< .0001
	Average	2.5	(2.1, 3.1)	
	Little	5.8	(4.8, 7.1)	
Father’s body shape	Average	1.0		< .0001
	Slim	1.0	(0.9, 1.2)	
	Obese	1.3	(1.2, 1.5)	
Mother’s body shape	Average	1.0		< .001
	Slim	1.2	(1.0, 1.4)	
	Obese	1.3	(1.1, 1.5)	

*OR* odds ratio; *CI* confidence interval.

Parameters entered into model: Gender, education, economic status, father’s job, parental interest in weight, father’s body shape, mother’s body shape.

**Table 4:** Adjusted odds ratio from multivariable logistic regression for childhood and adolescent overweight by gender

**Variable**	**Category**	**Male**			**Female**		
		**OR**	**95% CI**	***P*-value**	**OR**	**95% CI**	***P*-value**
Education	Senior secondary school	1.0		< .0001	1.0		< .0001
	Elementary	1.5	(1.2, 1.7)		2.6	(2.1, 3.2)	
Economic status	Upper				1.0		0.03
	Middle				1.0	(0.8, 1.2)	
	Lower				1.4	(1.0, 1.9)	
Parental interest in weight	Very much	1.0		< .0001	1.0		< .0001
	Average	2.7	(2.1, 3.4)		2.2	(1.4, 3.5)	
	Little	6.1	(4.9, 7.7)		5.8	(3.8, 8.9)	
Father’s body shape	Average	1.0		0.12	1.0		< .0001
	Slim	1.1	(0.9, 1.4)		0.9	(0.7, 1.2)	
	Obese	1.2	(1.0, 1.4)		1.5	(1.2, 1.9)	
Mother’s body shape	Average	1.0		< .001	1.0		0.01
	Slim	1.3	(1.1, 1.6)		0.9	(0.7, 1.2)	
	Obese	1.4	(1.2, 1.6)		1.3	(1.1, 1.7)	

*OR* odds ratio; *CI* confidence interval.

The male model included education, parental interest in weight, father’s body shape, mother’s body shape as independent variables.

The female model included education, economic status, parental interest in weight, father’s body shape, mother’s body shape as independent variables.

## Discussion

In this study, data from NYPI’s obesity status survey of 2009 were used to find the association between parental SES and the overweight status in children and adolescents. The prevalence of obesity in children and adolescents was higher when subjective household economic status was lower. And the father was unemployed, the risk of obesity could be increased (*P* = 0.084). The theoretical framework proposed by Sobal [16] acknowledges that each SES indicator may operate via a different pathway to influence the development of adiposity. According to this framework, education influences knowledge and beliefs, occupation influences lifestyle and shared peer values, and income relates to access to resources [9]. Therefore, economic status could affect their children’s access to resources for the healthy life style. Also, compared to employed fathers, children and adolescents of unemployed fathers showed higher rates of overweight.

This study, which used nationally representative data of Korea, reconfirms that the relationship between overweight and SES among Korean children and adolescents is consistent with findings from developed countries, where the prevalence of overweight is inversely associated to SES [18-20]. In the 2009/2010 study of Health Behavior in School-aged Children (HBSC), 13 out of 39 countries showed a statistically significant association between the low family affluence scale (FAS) and the overweight status in both genders. Moreover, another eight countries showed an inverse relationship between overweight and FAS in females. Lastly, the findings of this study also correspond with Korean adult women’s overweight patterns [21].

### Objective and subjective SES indicators for adolescents

In a study of the health equity of adolescents, the appropriateness of conventional indicators such as parental education level and household income was debated and various alternative indicators were proposed [22,23]. They stemmed from the particular characteristics of adolescents during the middle stages of the maturation process and the possibility of inaccurate measurement of parental SES by adolescents. For the latter, the FAS was created to be a more accurate indicator [22]. For the former, measurement of the SES of adolescents by asking their own status instead of parental SES was proposed [24,25]. Because adolescent SES was self-measured, the measure indicated the subjective SES of adolescents. Therefore, the debate on appropriate indicators of adolescent SES is interconnected with the origin of health inequality. If material deficiency is a major factor in health inequality, then an objective SES indicator such as FAS or well-measured parental SES is more strongly related to health outcomes. However, if psychosocial stress is a major factor, then subjective SES is more strongly related to health outcomes. In this analysis, objective SES and subjective SES indicators were both considered. Education level of parents and employment status could be regarded as the objective SES indicator. On the contrary, the subjective household economic status is reported by adolescents, and therefore it could be considered as the subjective SES reported by adolescents [19].

In this study, the results were inconsistent. Father’s employment status had a tendency related to overweight in children and adolescents when male and female students were considered together, which suggests that traditional indicators could be appropriate to study adolescents. However, subjective economic status had a significant effect on overweight also. And in subgroup analysis, economic status was associated with females being overweight. Therefore, objective SES and subjective SES had a different effect on each gender. In previous studies, it is proposed that objective SES, subjective SES and overweight status affect each other [26]. For example, objective SES has an effect on overweight through diet, physical activity or stress. Furthermore, subjective SES has an effect on overweight through stress and psychological sequelae such as social isolation. Also overweight results in both objective SES through stigma and discrimination and subjective SES through cultural norms and stigma. Therefore, it is appropriate to consider the various SES indicators including objective and subjective SES indicator because both could affect health outcomes independently [24,26].

### Biological characteristics of parents and the role of interest on adolescent’s overweight

Overweight patterns by gender were also related to the body shapes of parents. In the case of male students, when their mother was slender or obese, the risk of children and adolescent’s being overweight was higher. But in the case of female students, the risk of being overweight increased only when the mother was obese. Additionally, the father’s body shape had a significant effect on female students but not on male students. Therefore, differences in overweight inequality may result from gender. For example, dietary and physical inactivity that are important to overweight differ between the two genders [27].

Parental interest in weight management plays a significant role in adolescents through role modeling and encouraging healthy weight management. Studies exploring the relationship between parental interest in weight management, and adolescent diet or risk for overweight have been controversial. Authoritarian or disengaged parenting styles were consistently associated with a greater increase in adolescent BMI compared to balanced parenting styles [17], while other studies found no association between parental interest in weight management and BMI trajectories through childhood and adolescence [28,29]. In this study, less parental interest is related to high risk of overweight. But in this cross sectional study, it is hard to determine if parental less interest in weight is a cause for overweight in children.

The overweight or underweight status of parents could affect the overweight in children or adolescents by genetic traits or family life styles. In this study, when the parents were obese or slender, the odds of children and adolescents being overweight increased compared to when the parents were of average weight. However, because the overweight of parents was measured by children and adolescents, this measurement could have a bias. This result illustrates the inheritance of overweight by the subsequent generation. Therefore, appropriate interventions deterring the inheritance of overweight are needed. Because lifestyle may also be affected by SES, a targeted intervention for families with lower SES could be beneficial.

### Strength and weaknesses of this study

A previous study which used the 2007 Korea Youth Risk Behavior Web-based Survey, another nationally representative survey, showed an inverse relationship between overweight status and subjective social status [19]. Among SES indicators, subjective family economic status and subjective school achievement were statistically significant in multiple logistic regression analyses. This study also showed a negative relationship between SES and overweight in spite of differences in measurement methods and data source. In comparison to a previous study, elementary school children from 4th to 6th grade were included and the health outcome was defined as being overweight instead of obese, and the definition of being overweight changed. More importantly, the biologic factors of parents, which were not considered in a previous study, were also considered. In spite of these differences, the importance of SES was acknowledged. But there is a need for further studies on the appropriate SES indicators for adolescents.

The present study has some limitations. Firstly, because the present study is a cross-sectional study, causality could not be established. Although a previous cohort study also found evidence that SES was associated with overweight, the cross sectional design of the present study weakens the causality of overweight. Therefore, a longitudinal study that could further determine cause-outcome relationships is necessary. Another limitation is the inaccuracy of self-administered questionnaires and the lack of validation in the assessment of questions. In particular, overweight status was defined by self-reported height and weight. However, students are well known about their height and weight based on their physical examination that is mandatorily taken every year at the school in Korea. Children and adolescents may underestimate their obese status and the prevalence of overweight could be underestimated. Parent body shape variables are also reported by children. This could result in over or underestimation of association between children and parent BMI. Similarly, because the employment status is only classified as employed or unemployed, the detailed employment situation, such as having a professional job or an unskilled job cannot be ascertained. These aspects could also have an impact on the examination of the relationship between overweight and SES. These limitations pointed out the need for careful interpretation of the findings in this study and future research on adolescent overweight.

## Conclusions

This study showed an inverse association between overweight and SES in Korean children and adolescents through an examination of nationally representative data. The prevalence of overweight was higher in the low economic status group. Also, in a subgroup analysis by gender, which adjusted for demographic factors and SES indicators, subjective family economic status, and paternal education level had significant effects on the overweight status of children and adolescents. These results match previous findings on SES-overweight inverse patterns in Korean adolescents in spite of difference in measurement methods, time, and data sources. This confirms that the overweight pattern of Korean children and adolescents matches that of other developed countries. An appropriate intervention targeting the inheritance of overweight should be an area of concern in Korea.

## Abbreviations

SES: Socioeconomic status; OR: Odds ratio; CI: Confidence interval; BMI: Body mass index; NYPI: National Youth Policy Institute; IOTF: International Overweight Task Force; HBSC: Health Behavior in School-aged Children; FAS: Family affluence scale; GDP: Gross domestic product; SD: Standard deviation.

## Competing interests

The authors declare that they have no competing interests.

## Authors’ contributions

JW participated in the design of the study, interpretation of data, and drafting the manuscript.YE made substantial contributions to the analysis and interpretation of data. IH was involved in drafting the manuscript and revising it critically for important intellectual content. YD was accountable for all aspects of the work in ensuring that questions related to the accuracy, design or integrity of this paper. All authors read and approved the final manuscript.

## Pre-publication history

The pre-publication history for this paper can be accessed here:

http://www.biomedcentral.com/1471-2458/14/324/prepub

## References

[B1] National Center for Health Statistics: Health, United States With Special Features on Socioeconomic Status and Health 2011 Hyattsville, MD: U.S. Department of Health and Human Services. 2012 22812021

[B2] Ogden CL Carroll MD Kit BK Flegal KM Prevalence of obesity and trends in body mass index among US children and adolescents, 1999–2010 JAMA 2012 307 483 490 10.1001/jama.2012.40 22253364 PMC6362452

[B3] Oh K Jang MJ Lee NY Moon JS Lee CG Yoo MH Kim YT Prevalence and trends in obesity among Korean children and adolescents in 1997 and 2005 Korean J Pediatr 2008 51 950 955 10.3345/kjp.2008.51.9.950

[B4] Sato AF Jelalian E Hart CN Lloyd-Richardson EE Mehlenbeck RS Neill M Wing RR Associations between parent behavior and adolescent weight control J Pediatr Psychol 2011 36 451 460 10.1093/jpepsy/jsq105 21112925 PMC3079126

[B5] McLaren L Socioeconomic status and obesity Epidemiol Rev 2007 29 29 48 10.1093/epirev/mxm001 17478442

[B6] Gibson LY Byrne SM Davis EA Jacoby P Zubrick SR The role of family and maternal factors in childhood obesity Med J Aust 2007 186 591 595 17547550 10.5694/j.1326-5377.2007.tb01061.x

[B7] Wang Z Patterson CM Hills AP Association between overweight or obesity and household income and parental body mass index in Australian youth: analysis of the Australian national nutrition survey, 1995 Asia Pac J Clin Nutr 2002 11 200 205 10.1046/j.1440-6047.2002.00291.x 12230233

[B8] Lake JK Power C Cole TJ Child to adult body mass index in the 1958 British birth cohort: associations with parental obesity Arch Dis Child 1997 77 376 381 10.1136/adc.77.5.376 9487953

[B9] Svensson V Jacobsson JA Fredriksson R Danielsson P Sobko T Schio HB Marcus C Associations between severity of obesity in childhood and adolescence, obesity onset and parental BMI: a longitudinal cohort study Int J Obes (Lond) 2011 35 46 52 10.1038/ijo.2010.189 20856258 PMC3035977

[B10] Shrewsbury V Wardle J Socioeconomic status and adiposity in childhood: a systematic review of cross-sectional studies 1990–2005 Obesity 2008 16 275 284 10.1038/oby.2007.35 18239633

[B11] Hanson MD Chen E Socioeconomic status and health behaviors in adolescence: a review of the literature J Behav Med 2007 30 263 285 10.1007/s10865-007-9098-3 17514418

[B12] Ki M Choi BY Kim MK Fang JN Xu CY Ahn DH Kang YJ Relationship between adolescent obesity and socioeconomic status of parents: in Seoul, Yangpyong, and Yanbian area Korean J Prev Med 1999 32 9 16

[B13] Kim J Im J Yim J Park SH Hong DH The relationship between economic status and adolescent obesity in Incheon, Korea Korean J Obes 2007 16 76 85

[B14] NYPI Cross sectional survey data 2009 http://archive.nypi.re.kr/sub.asp?BID=B32&idx=700&BoardType=view&page=1&Search_m=&Search_t=&Mcode=B020090 (accessed 3 March 2013)

[B15] International Association for the Study of Obesity Extended international (IOTF) body mass index cut-offs for thinness, overweight and obesity in children http://www.iaso.org/resources/aboutobesity/child-obesity/newchildcutoffs (accessed 3 September 2012) 10.1111/j.2047-6310.2012.00064.x 22715120

[B16] Sobal J Obesity and socioeconomic status: a framework for examining relationships between physical and social variables Med Anthropol 1991 13 231 247 10.1080/01459740.1991.9966050 1961104

[B17] Fuemmeler BF Yang C Costanzo P Hoyle RH Siegler IC Williams RB Ostbye T Parenting styles and body mass index trajectories from adolescence to adulthood Health Psychol 2012 31 441 449 22545979 10.1037/a0027927 PMC3616616

[B18] Haug E Rasmussen M Samdal O Iannotti R Kelly C Borraccino A Vereecken C Melkevik O Lazzeri G Giacchi M Ercan O Due P Ravens-Sieberer U Currie C Morgan A Ahluwalia N HBSC Obesity Writing Group Overweight in school-aged children and its relationship with demographic and lifestyle factors: results from the WHO-collaborative health behaviour in school-aged children (HBSC) study Int J Public Health 2009 54 Suppl 2 167 179 19618111 10.1007/s00038-009-5408-6 PMC2735089

[B19] Oh IH Cho Y Park SY Oh C Choe BK Choi JM Yoon TY Relationship between socioeconomic variables and obesity in Korean adolescents J Epidemiol 2011 21 263 270 10.2188/jea.JE20100099 21532240 PMC3899418

[B20] Currie C Zanotti C Morgan A Currie D De Looze M Roberts C Samdal O Smith RF Barnekow V Social Determinants of Health and Well-Being Among Young People Health Behaviour in School-Aged Children (HBSC) Study 2012 Copenhagen: WHO Regional Office for Europe

[B21] Yoon YS Oh SW Park HS Socioeconomic status in relation to obesity and abdominal obesity in Korean adults: a focus on sex differences Obesity (Silver Spring) 2006 14 909 919 10.1038/oby.2006.105 16855201

[B22] Currie CE Elton RA Todd J Platt S Indicators of socioeconomic status for adolescents: the WHO health behaviour in school-aged children survey Health Educ Res 1997 12 385 397 10.1093/her/12.3.385 10174221

[B23] West P Sweeting H Evidence on equalisation in health in youth from the West of Scotland Soc Sci Med 2004 59 13 27 10.1016/j.socscimed.2003.12.004 15087139

[B24] Goodman E Adler NE Kawachi I Frazier AL Huang B Colditz GA Adolescents’ perceptions of social status: development and evaluation of a new indicator Pediatrics 2001 108 e31 10.1542/peds.108.2.e31 11483841

[B25] Piko B Fitzpatrick KM Does class matter? SES and psychosocial health among Hungarian adolescents Soc Sci Med 2001 53 817 830 10.1016/S0277-9536(00)00379-8 11511056

[B26] Goodman E Adler NE Daniels SR Morrison JA Slap GB Dolan LM Impact of objective and subjective social status on obesity in a biracial cohort of adolescents Obes Res 2003 11 1018 1026 10.1038/oby.2003.140 12917508

[B27] Simen-Kapeu A Veugelers PJ Should public health interventions aimed at reducing childhood overweight and obesity be gender-focused? BMC Public Health 2010 10 340 10.1186/1471-2458-10-340 20546619 PMC2894777

[B28] Gable S Lutz S Household, parent, and child contributions to childhood obesity Fam Relat 2000 49 293 300 10.1111/j.1741-3729.2000.00293.x

[B29] Agras WS Hammer LD McNicholas F Kraemer HC Risk factors for childhood overweight: a prospective study from birth to 9.5 years J Pediatr 2004 145 20 25 10.1016/j.jpeds.2004.03.023 15238901

